# Linkage of methionine addiction, histone lysine hypermethylation, and malignancy

**DOI:** 10.1016/j.isci.2022.104162

**Published:** 2022-03-25

**Authors:** Jun Yamamoto, Sachiko Inubushi, Qinghong Han, Yoshihiko Tashiro, Norihiko Sugisawa, Kazuyuki Hamada, Yusuke Aoki, Kentaro Miyake, Ryusei Matsuyama, Michael Bouvet, Steven G. Clarke, Itaru Endo, Robert M. Hoffman

**Affiliations:** 1 AntiCancer Inc, 7917 Ostrow St, San Diego, CA 92111, USA; 2 Department of Surgery, University of California, San Diego, 9300 Campus Point Drive #7220, La Jolla, CA 92037-7220, USA; 3 Department of Gastroenterological Surgery, Yokohama City University Graduate School of Medicine, 3-9 Fukuura, Kanazawa-ku, Yokohama 236-0004, Japan; 4 Department of Chemistry and Biochemistry, University of California, Los Angeles, Los Angeles, CA 90095-1569, USA

**Keywords:** Biological sciences, Molecular biology, Cell biology, Cancer

## Abstract

Methionine addiction, found in all types of cancer investigated, is because of the overuse of methionine by cancer cells for excess transmethylation reactions. In the present study, we compared the histone H3 lysine-methylation status and degree of malignancy between methionine-addicted cancer cells and their isogenic methionine-independent revertants, selected by their growth in low concentration of methionine. The methionine-independent revertans can grow on low levels of methionine or independently of exogenous methionine using methionine precursors, as do normal cells. In the methionine-independent revertants, the excess levels of trimethylated histone H3 lysine marks found in the methionine-addicted parental cancer cells were reduced or lost, and their tumorigenicity and experimental metastatic potential in nude mice were also highly reduced. Methionine addiction of cancer is linked with malignancy and hypermethylation of histone H3 lysines. The results of the present study thus provide a unique framework to further understand a fundamental basis of malignancy.

## Highlights

• Methionine(MET)-independent revertants were selected from MET-addicted cancer cells
• MET-independent revertants had greatly reduced malignancy
• MET-independent revertants have lost or reduced methylation of H3 lysine marks
• MET addiction, malignancy, and hypermethylated H3 lysine marks are linked

## Graphical abstract

**Figure:**
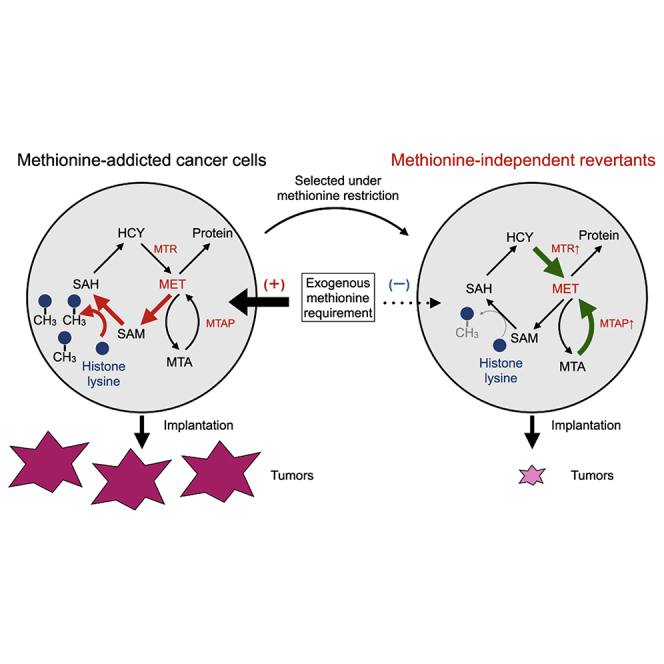

## Introduction

Since the time of Warburg more than 100 years ago, the relationship of metabolism and cancer has been studied.

Methionine addiction is a fundamental and general hallmark of cancer and is known the Hoffman effect (Coalson et al., 1982; Hoffman and Erbe, 1976; Kaiser, 2020; Stern and Hoffman, 1984; Stern et al., 1983, 1984) and is an area of current intense interest (Gao et al., 2019; Wang et al., 2019; Yamamoto et al., 2020a, 2021, 2022). Methionine addiction is characterized by a requirement for exogenous methionine for growth by cancer cells even though the methionine-addicted cancer cells synthesize normal or excess amounts of methionine (Coalson et al., 1982; Hoffman and Erbe, 1976; Stern and Hoffman, 1984; Stern et al., 1983; Wang et al., 2019). Methionine restriction (MR) by either methionine-free medium (Hoffman and Jacobsen, 1980) or by a low-methionine diet *in vivo* (Guo et al., 1993) or by methioninase (Yano et al., 2014) selectively arrests cancer cells in the late S/G_2_ phase of the cell cycle, but not normal cells, where the cancer cells become selectively sensitive to cytotoxic chemotherapy (Gao et al., 2019; Hoshiya et al., 1995, 1997; Stern et al., 1984; Tan et al., 1999; Yano et al., 2014; Yoshioka et al., 1998). Methionine addiction results from the overuse of methionine by cancer cells for excess transmethylation reactions (Judde et al., 1989; Stern and Hoffman, 1986; Wang et al., 2019). The Hoffman effect of methionine addiction is analogous to the Warburg effect for glucose overuse by cancer cells (Borrego et al., 2016; Kaiser, 2020). Methionine addiction is tightly linked to other hallmarks of cancer (Borrego et al., 2016; Hoffman et al., 1979; Yamamoto et al., 2020a, 2021, 2022) and is thought possibly the very basis of malignancy itself (Hoffman, 2017).

Although we have long since known methionine is overused for transmethylation reactions in cancer cells (Judde et al., 1989; Stern and Hoffman, 1984), we have poorly understood the fate of at least a significant amount of the excess methyl groups that were transferred. Our previous studies have shown that histone H3 lysine marks are overmethylated in cancer cells compared to normal cells (Aoki et al., 2021; Yamamoto et al., 2020a, 2021, 2022) and that the histone H3 lysine hypermethylation is unstable during methionine restriction of methionine-addicted cancer cells which arrests their proliferation (Yamamoto et al., 2020a). In contrast, a lesser amount of histone H3 lysine methylation is stable in normal cells under MR, which unlike cancer cells do not arrest their proliferation under methionine restriction (Hoffman and Erbe, 1976; Yamamoto et al., 2020a). These results suggested that histone H3 lysine hypermethylation may be related to methionine addiction.

In the present study, we report that isogenic methionine-independent revertants, selected from parental methionine-addicted cancer cells, by methionine restriction with recombinant methioninase, lose hypermethylation at histone H3 lysine marks and have greatly reduced malignancy compared to their methionine-addicted parental cancer cells and lose their requirement for exogenous methionine. Our results indicate that histone H3 lysine hypermethylation is closely associated with methionine addiction and malignancy, suggesting a possible causal relationship.

## Results

### Methionine-addicted cancer cells selected to grow in low-methionine media revert to methionine independence

**Figure 1:**
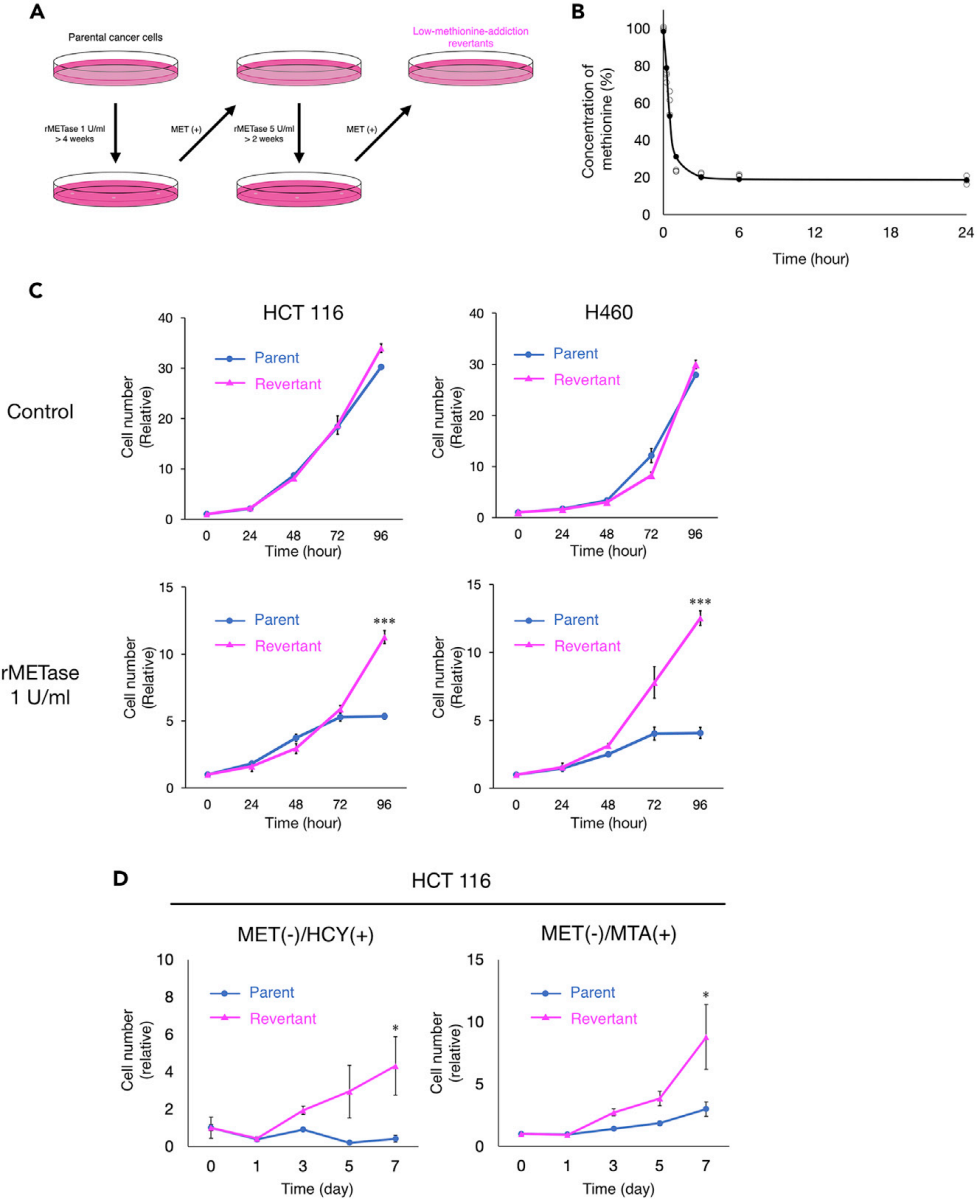
Comparison of sensitivity to methioninase of methionine-addicted cancer cells and their isogenic methionine-independent revertants *in vitro* (A) Diagram of the establishment of low-methionine-requirement (methionine-independent) revertants. (B) Recombinant methioninase (rMETase) was used to deplete methionine in the cell culture medium. rMETase was added to the medium (1 U/mL) and the methionine level in the medium was measured at 0 h, 15 min, 30 min, 1 h, 3 h, 6 h, and 24 h (n = 3). (C) Cell proliferation assay of methionine-addicted parental cancer cells and their isogenic methionine-independent revertants under methionine restriction by rMETase. Cells were cultured in methionine-containing medium or methionine-containing medium with rMETase (1 U/mL) for 24, 48, 72, and 96 h (mean ± SEM, n = 3. ∗∗∗, p < 0.001, Student’s *t* test). (D) Cell proliferation assay of methionine-addicted parental cancer cells and their isogenic methionine-independent revertants in methionine-free medium containing 200 μM DL-homocysteine or 250 nM methylthioadenosine (MTA) with 10% dialyzed fetal bovine serum (mean ± SEM, n = 3. ∗, p < 0.05, Student’s *t* test).

We used recombinant methioninase (rMETase) (Hoffman, 2015) to deplete methionine *in vitro*. The level of methionine in the medium was rapidly decreased to less than 30% within 1 h after addition of 1 U/mL of rMETase (Figure 1B).

To compare the methionine requirement of methionine-addicted parental cancer cells and isogenic low-methionine-requirement revertants derived from the parental cells, we then evaluated their cell proliferation kinetics under normal methionine conditions and methionine restriction affected by rMETase. The proliferation of methionine-low-requirement revertants was not significantly different from that of methionine-addicted cancer cells under normal methionine conditions *in vitro* (HCT116, p = 0.063; H460, p = 0.072, respectively) (Figure 1C). The proliferation of parental methionine-addicted cancer cells arrested within 72–96 h of methionine restriction. In contrast, methionine-low-requirement revertants were able to continuously proliferate with a significant difference from their methionine-addicted cancer cells under methionine restriction (HCT116, p < 0.001; H460, p < 0.001, respectively), growing similar to normal cells (Borrego et al., 2016; Hoffman et al., 1978, 1979; Judde et al., 1989) (Figure 1C). We also evaluated the cell proliferation of low methionine-requirement revertants in methionine-free medium supplemented with dialyzed fetal bovine serum containing homocysteine or methylthioadenosine (MTA). Low-methionine-requirement revertants proliferated in a medium in which methionine was replaced by either homocysteine or MTA similar to normal cells (Figure 1D). In contrast, parental methionine-addicted cancer cells arrested in both replacement media (homocysteine, p = 0.040; MTA, p = 0.049, respectively). These results indicate that the revertants have lost their methionine addiction and requirement for exogenous methionine and are termed methionine-independent.

### Methionine synthase and methylthioadenosine phosphorylase are upregulated in methionine-independent revertants

**Figure 2:**
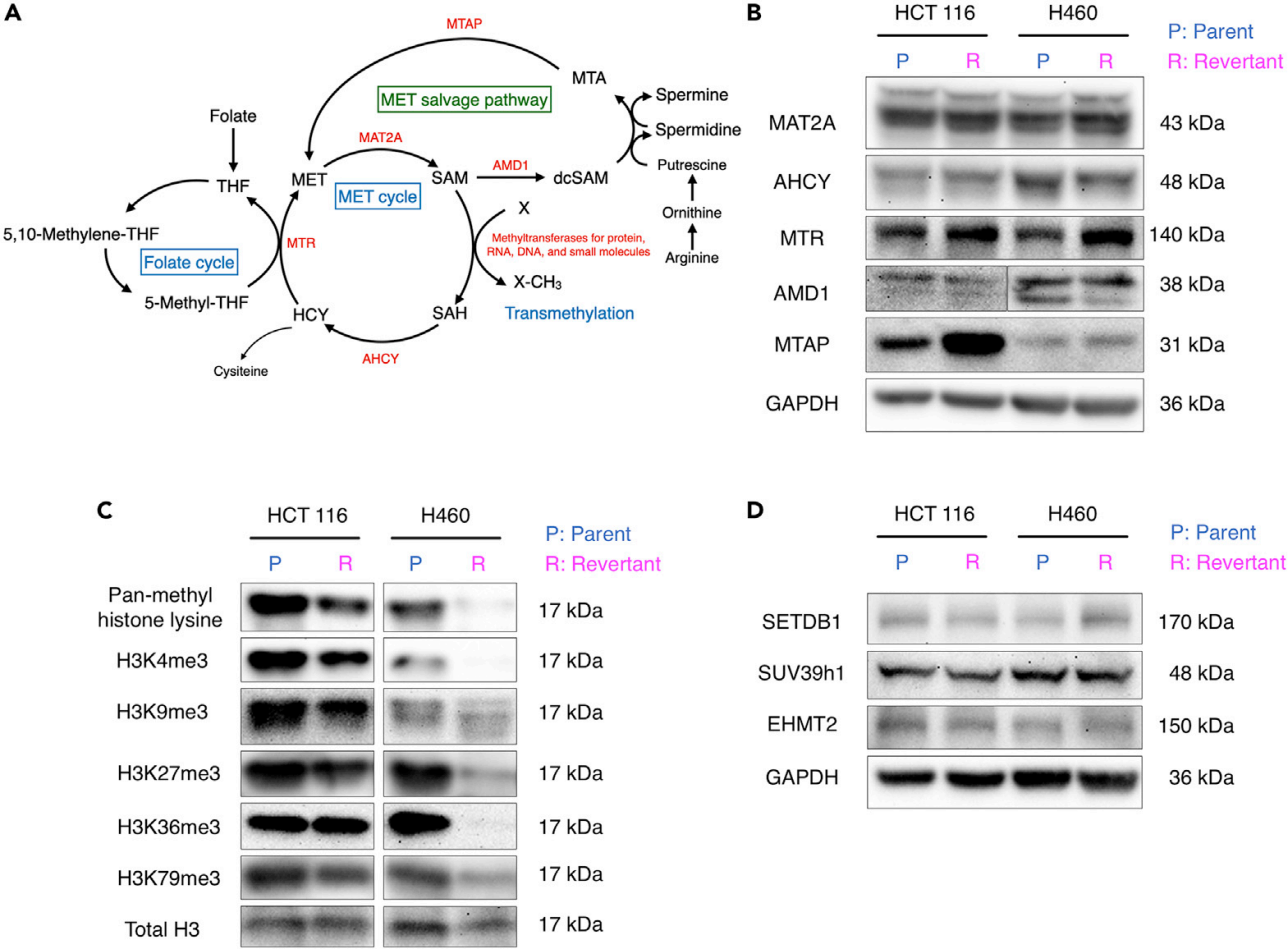
Methionine and methyl group metabolism in parental methionine-addicted cancer cells and their isogenic methionine-independent revertants *in vitro* (A) The pathways of methionine metabolism. (B) Immunoblot of the enzymes related to methionine metabolism. (C) Immunoblot of pan-methyl H3 lysine methylation and trimethylated histone H3 lysine marks in methionine-addicted cancer cells and methionine-independent revertants grown in methionine-containing medium. (D) Immunoblot of H3K9 methyltransferases in methionine-addicted cancer cells and isogenic methionine-independent revertants grown in methionine-containing medium.

To determine the differences in methionine metabolism in methionine-addicted cancer cells and methionine-independent revertants, we examined changes in protein expression levels for enzymes involved in methionine biosynthesis and methionine salvage in parental methionine-addicted HCT 116 and H460 cells and their respective isogenic methionine-independent revertants (Figure 2A). We found methionine synthase (MTR) was modestly upregulated in methionine-independent revertants of each cell line and that methylthioadenosine phosphorylase (MTAP) was strongly upregulated in the HCT 116 revertant cells and moderately upregulated in the H460 revertant cells (Figure 2B).

### Hypermethylation of histone H3 lysine marks is strongly decreased in methionine-independent revertants

We then compared the methylation status of histone H3 lysine marks in methionine-addicted parental cells and methionine-independent revertants. We have previously shown that methionine-addicted cancer cells have an excess of histone H3 lysine-mark methylation compared to normal fibroblasts. Parental cancer cells and methionine-independent revertants were cultured in normal methionine-containing medium for 96 h and histones were extracted. The overall level of lysine methylation of histone H3 was decreased in the methionine-independent revertants compared to parental methionine-addicted cells, even in the presence of methionine (Figure 2C). The levels of trimethylated histone H3 lysine marks, including H3K4me3, H3K9me3, H3K27me3, H3K36me3, and H3K79me3, were all decreased in the methionine-independent revertants compared to their parental methionine-addicted cells in the presence of the methionine (Figure 2C). However, the expression levels of several histone methyltransferase enzymes, which methylate H3K9me3, did not change in the methionine-independent revertants (Figure 2D).

These results show that methionine addiction and histone H3 lysine-mark hypermethylation are closely associated.

### Methionine-independent revertants lose malignancy

**Figure 3:**
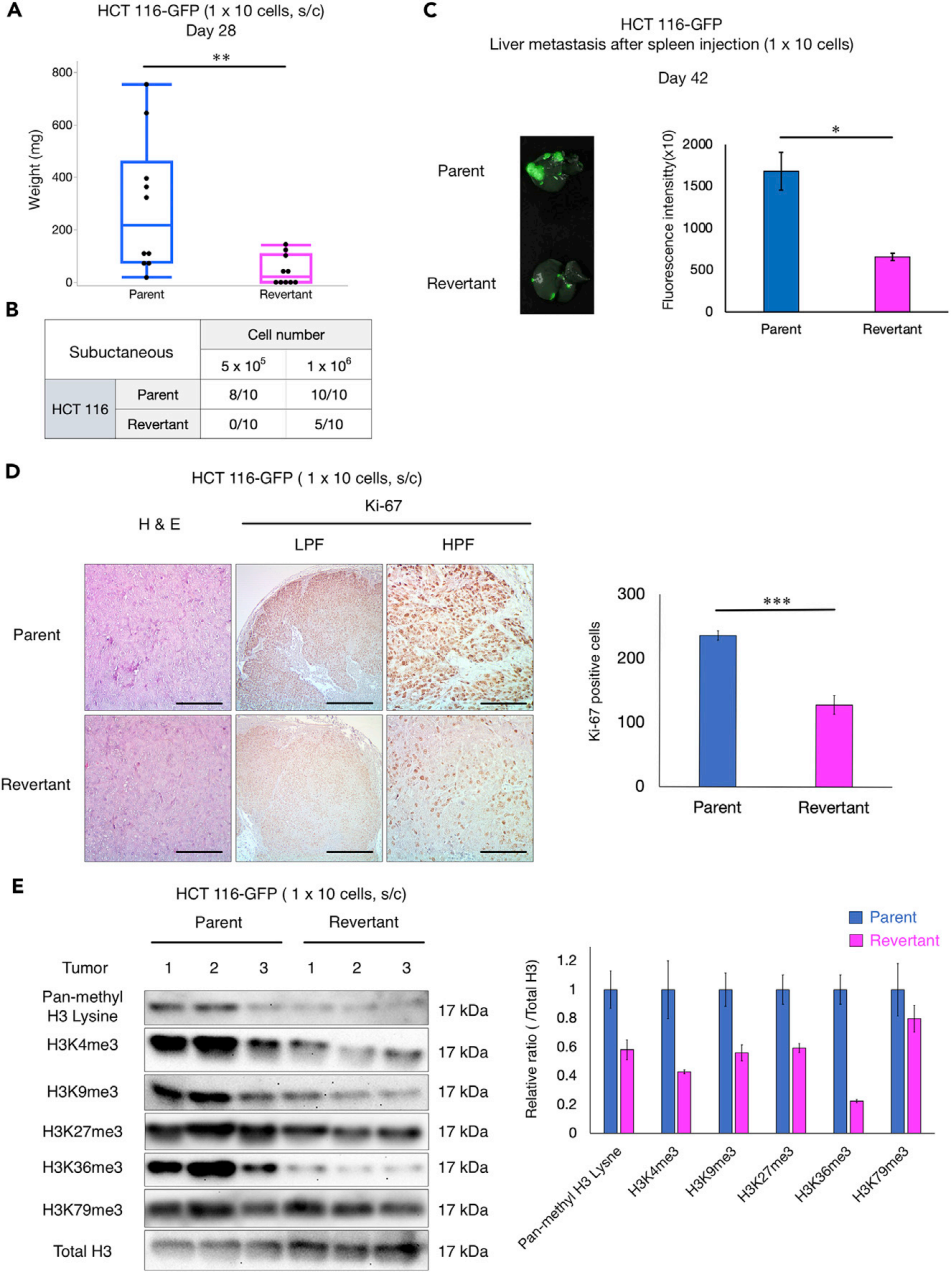
Comparison of malignancy of methionine-addicted cancer cells and their isogenic methionine independent revertants *in vivo* (A) Mean tumor weight of HCT 116 and HCT 116-R at 28 days after 1 × 10^6^ cells were injected in nude mice (mean ± SD, n = 10. ∗∗, p < 0.01, Student’s *t* test). (B) Number of the tumors in nude mice with tumors at day 28 of HCT 116 and HCT 116-R. (C) Experimental liver metastasis: parental methionine-addicted HCT 116-GFP and methionine-independent revertants HCT 116-R-GFP. Left: representative fluorescence image of liver metastasis. Right: fluorescence intensity (mean ± SD, n = 3. ∗, p < 0.05, Student’s *t* test). (D) Left: Representative images of H&E staining and immunohistochemical staining for Ki-67 of HCT 116 and HCT 116-R subcutaneous tumors. LPF: low power field (40×). HPF: high power field (200×). Scale bar: 100 μm (H&E and HPF of Ki-67), 500 μm (LPF of Ki-67). Right: Quantification of the Ki-67-positive cells. Five high-power fields were randomly chosen (400×) and positive cells compared between parent and revertant cells (mean ± SEM, n = 4. ∗∗∗, p < 0.001, Student’s *t* test). (E) Left: Immunoblot of pan-methyl H3 lysine and trimethyl histone H3 marks in subcutaneous tumors formed from HCT 116 and HCT 116-R (n = 3). Right: The ratio of pan-methyl H3 lysine and trimethyl-histone H3 marks/total H3 in the tumors formed from HCT 116 and HCT 116-R (mean ± SEM, n = 3).

To compare the malignancy of parental methionine-addicted cancer cells and isogenic methionine-independent revertants derived from them, the tumorigenicity of the parental and revertant cells was compared in subcutaneous-xenograft mouse models. Methionine-addicted parental HCT 116 cells and their methionine-independent revertants (HCT 116-R) were compared for their ability to form tumors in female nude mice. The mean tumor volume was significantly lower in HCT 116-R tumors than HCT 116 tumors after injection of 1 × 10^6^ cells in nude mice (p = 0.0085) and only half of 10 mice formed tumors in HCT 116-R compared to all mice with HCT 116 (Figure 3A). Although 8 out of 10 mice injected with 5 × 10^5^ parental methionine-addicted HCT 116 cells formed tumors, no mice injected with 5 × 10^5^ HCT 116-R cells formed tumors. This is consistent with our previous study that showed methionine-independent revertants of H460 cells also lost tumorigenicity (Yamamoto et al., 2021). These results also show that, *in vivo* under methionine-replete conditions, the revertants grow much less than their parental methionine-addicted cells.

The metastatic potential of HCT 116-GFP expressing cells and HCT116-R-GFP expressing cells was also compared after spleen injection of the two cell types in female nude mice. Spleen injection of cancer cells is used to determine their capacity to form experimental liver metastasis (Bouvet et al., 2006). The parental HCT 116-GFP cells formed significantly more experimental liver metastasis compared to revertant HCT-116-R-GFP cells (p = 0.011) (Figure 3B). These results show that methionine-independent revertants have significantly reduced malignancy.

Immunohistochemistry staining showed that the number of Ki-67-positive cells was significantly lower in the HCT 116-R subcutaneous tumors compared to the parental HCT 116 subcutaneous tumors (n = 4, p < 0.0001), indicating reduced *in vivo* proliferation of the methionine-independent revertants under methionine-replete conditions (Figure 3C). The tumorigenicity, metastasis, and cell proliferation results indicate that methionine-independent revertants are much less malignant than their isogenic methionine-addicted parents. Although methionine-addicted cancer cells and their isogenic methionine-independent revertants have similar growth potential in normal methionine conditions *in vitro*, the methionine-independent revertants have greatly reduced growth *in vivo*.

### RNA sequencing of methionine-addicted parent and methionine-independent revertant colon cancer and lung cancer cell lines shows no large-scale differences in gene expression

To determine if large-scale changes in gene expression are involved in the reversion of cancer cell lines from methionine-addiction to methionine-independence and loss of tumorigenicity, we compared mRNA transcript levels in parent and methionine-addicted cancer cells and their methionine-independent revertant cells from the HCT 116 cell line and the H460 cell line. In Tables S1 and S2, we show genes with transcription levels that were significantly elevated or depressed more than twofold between the parent and revertant cell lines. Of the some 19,000 human genes measured for expression, only a small number met these criteria. For the HCT 116 parental cells, we found that 14 genes were downregulated at least by twofold and 7 genes were upregulated by at least twofold, compared to HCT 116 revertant cells (Table S1). The corresponding numbers for the H460 cells were 80 genes downregulated and 14 upregulated genes in the revertants compared to parental cells (Table S2). We found no common genes whose expression changed twofold or more in the revertants compared to their parents in H460 and HCT 116 cells. These results suggest that there may be no common transcriptional pathway that would account for the reversion of methionine-addicted cancer cells to methionine-independence, or the methods used were not sensitive enough to detect such common genes. With the possible exception of the FOLR1 gene encoding the alpha folate receptor, none of these genes with a twofold or more change appeared to be involved in methionine metabolism.

### Changes in expression of genes involved in methionine metabolism in methionine-addicted parental cancer cells compared with methionine-independent revertants

We then examined the changes in gene expression for genes involved in the biosynthesis of methionine, its conversion to *S*-adenosylmethionine, and the use of *S*-adenosylmethionine for polyamine biosynthesis and transmethylation reactions, as well as genes encoding enzymes involved in demethylation when the cancer cells reverted from methionine-addiction to methionine-independence (Figure 2B, Tables 1, S1, S2, and S3). These genes included methionine synthase and related proteins, *S*-adenosylmethionine synthetase, enzymes and proteins of the folate cycle that provide methyl groups for methionine biosynthesis, methionine salvage and polyamine biosynthetic proteins, and enzymes involved in the metabolism of the product of all methyltransferases, *S*-adenosylhomocysteine. In addition, we examined the expression of genes for 182 putative and established *S*-adenosylmethionine-dependent methyltransferases, catalyzing methylation of proteins, RNA, DNA, and small molecules, as well as genes for 17 putative and established demethylases of protein, DNA, and RNA. We found very few changes in the expression of these genes when we compared the parents and revertants of the HCT 116 and H460 cancer cell lines, respectively. The most interesting statistically significant change was an almost two-fold increase in the expression of the MAT2A gene encoding the catalytic subunit of the S-adenosylmethionine synthetase in the H460 revertant (p = 0.01) (Table 1). However, the expression of this gene was decreased in the HCT 116 revertant cells. In addition, we should point out that changes in mRNA levels need not necessarily be correlated with changes in the activities of the protein, because translational and posttranslational controls may play larger roles than transcriptional controls in determining the activities of some enzymes. For example, when we looked at the protein levels of MAT2A by immunoblotting in Figure 2B, we observed no change in the methionine-independent revertants of either of the methionine-addicted cancer cell lines.

**Table 1:** Expression of genes related to methionine metabolism in methionine-addicted cancer cells and their methionine-independent revertants

Group	Protein	Gene name	HCT 116		H460	
			log2 Fold Change (revertant/parent)	p value	log2 Fold Change (revertant/parent)	p value
Methionine synthase and related proteins	Methionine synthase	MTR	0.00	0.99	−0.17	0.50
	Methionine synthase reductase	MTRR	−0.02	0.91	0.21	0.41
AdoMet synthetases		MAT1A	−1.50	0.27	0.70	0.66
		MAT2A	−0.52	0.31	0.93	0.01
	Regulatory subunit for MAT2A	MAT2B	0.09	0.49	0.14	0.57
Folate cycle enzymes that support methionine synthesis	Serine transhydroxylmethylase (cytoplasmic)	SHMT1	−0.04	0.76	−0.01	0.98
	Serine transhydroxymethyltransferase (mitochondrial)	SHMT2	0.04	0.73	−0.56	0.06
	methylene-THF reductase	MTHFR	−0.37	0.12	−0.27	0.40
	Folate receptors	FOLR1	−1.06	0.02		
	Thymidylate synthase	TYMS	0.18	0.19	0.29	0.28
Methionine salvage and polyamine biosynthesis	Methylthioadenosine phosphorylase	MTAP	0.04	0.74	0.08	0.74
	AdoMet decarboxylase	AMD1	−0.21	0.14	0.60	0.05
	Spermidine synthase	SRM	0.19	0.19	0.13	0.61
	Spermine synthase	SMS	0.10	0.45	0.06	0.79
AdoMet sensors		BMT2	0.27	0.22	−0.37	0.26
Homocysteine metabolism	Adenosylhomocysteine hydrolase	AHCY	0.04	0.73	−0.13	0.59
		AHCYL1	−0.13	0.35	−0.15	0.54
		AHCYL2	0.01	0.93	−0.52	0.08
	Cystathionine beta synthase	CBS			−0.30	0.50
		CBSL			−0.52	0.31
	Cystathionine gamma lyase	CTH	−0.15	0.50	−0.75	0.03
Glycine metabolism	Sarcosine dehydrogenase	SARDH	−0.31	0.22	0.58	0.59
	Dimethylglycine dehydrogenase	DMGDH	−0.12	0.88		

As noted above, we also found a statistically significant change in the expression of the gene for the alpha folate receptor in the HCT 116 revertants (∼two-fold decrease; p = 0.02) and the gene encoding an enzyme in the trans-sulfuration pathway from homocysteine to cysteine (cystathionine gamma lyase, which had a 1.7-fold decrease in the H460 revertants; p = 0.03). However, changes were not common to the revertants of both colon-cancer and lung-cancer cell lines (Table S4).

We noted statistically significant decreases in the expression of the genes in the revertant lines from both the HCT 116 and H460 cells for two protein lysine demethylases, KDM6B in the H460 revertant (1.9-fold, p = 0.04) and KDM7A in the HCT 116 revertant (1.5-fold; p = 0.02) (Table S3). However, from the immunoblotting results in Figure 2D, we observed decreases in the level of histone H3 trimethylation at this site in both H460 and HCT 116 revertant, indicating that the decrease in levels of histone H3 lysine methylation in the methionine-independent revertant is not because of demethylases but possibly because of decreased *S*-adenosylmethionine utilization.

The present results suggest that there are few significant changes in gene expression associated with reversion to a methionine-independent phenotype.

## Discussion

The present study shows that all measured trimethylated histone H3 lysine marks (as well as H3 pan methylation) that are in excess in methionine-addicted cancer cells compared to normal fibroblasts (Yamamoto et al., 2020a) are very reduced in their respective isogenic methionine-independent revertants. The revertants have greatly decreased malignancy, and unlike their respective parents, can proliferate under MR, or in the absence of exogenous methionine when it is replaced by homocysteine or MTA. The present study along with our recent study (Yamamoto et al., 2020a), suggest that malignant cells require histone H3 lysine hypermethylation to grow *in vitro* and to form tumors and metastasis *in vivo*. Our recent results have demonstrated that high malignancy variants of methionine-addicted parental cancer cells, become even more methionine-addicted, form large tumors, and increase the amount of trimethylated histone H3 lysine marks (Yamamoto et al., 2022), further emphasizing the close association of the three phenomena.

The present study thus shows that histone H3 pan-overmethylated lysine marks explain, at least in part, the fate of an important set of methyl-groups in cancer cells and why cancer cells are methionine addicted, but not normal cells or methionine-independent revertant cells. Most importantly, hypermethylation of histone H3 lysine residues is lost along with malignancy and methionine addiction in isogenic methionine-independent revertants, derived from methionine-addicted cancer cells. We have previously published that under methionine restriction by methioninase, H3K4me3 and H3K9me3 methylation is strongly reduced in methionine-addicted HCT116 and H460 cancer cells, during which their growth is arrested and viability reduced (Yamamoto et al., 2020a). Raboni et al. have confirmed our observation showing methioninase treatment of methionine-addicted cancer cell line caused a loss of the methylation of histone H3 lysine marks as well as growth arrest (Raboni et al., 2021). The present report thus demonstrates that methionine addiction, malignancy, and histone H3 hypermethylation are closely associated and possibly have a causal relationship, which is consistent with our previous studies (Hoffman et al., 1978, 1979; Yamamoto et al., 2020a, Yamamoto et al., 2021, 2022). Differences in histone H4 and other marks will be investigated in the future.

The first methionine-independent revertants isolated 44 years ago from methionine-addicted SV-40-transformed human fibroblasts (Hoffman et al., 1978, 1979) and recent methionine-independent revertants of triple negative breast cancer cells had reduced clonogenicity in agar, a surrogate marker of malignancy (Borrego et al., 2016), which gave the first hints that methionine addiction is linked to malignancy. Our present results further suggest hypermethylation of histone H3 lysine marks, including trimethyl histone H3 marks, may be necessary for malignancy as indicated by reduction in tumorigenicity, metastasis, and cancer cell proliferation within tumors of methionine-independent revertants, which is associated with greatly reduced methylation of histone H3 lysine methylation marks.

Consistent with the results of the present study, all tested cancer types are significantly more sensitive to methionine restriction by medium, diet, or methioninase than any tested normal cell type and are selectively arrested, compared to normal cells, by methionine restriction (Goseki et al., 1995; Guo et al., 1993; Hoffman, 2015; Hoffman and Jacobsen, 1980; Hoshiya et al., 1995; Kawaguchi et al., 2018b; Mecham et al., 1983; Tan et al., 1996, 1999, 2010; Yano et al., 2014).

We have previously shown that methionine-independent revertants isolated from methionine-addicted cancer cells have reverted to normal morphology, have reduced invasion capability, and greatly reduced ability to form colonies in soft agar (Hoffman et al., 1978; Hoffman et al., 1979; Yamamoto et al., 2022). The present study reemphasizes that global hypermethylation was observed in all of a large series of cell lines derived from multiple cancer types (Stern and Hoffman, 1984). Subsequently Judde et al. (Judde et al., 1989) observed in methionine-independent revertants isolated from methionine-addicted cancer cells that global transmethylation was greatly reduced. Wang et al. (2019) observed elevated methylation of histone H3 lysine marks in tumor-initiating cells relative to non-tumor-initiating cells. We also previously observed elevated H3K4me3 and H3K9me3 in methionine-addicted H460 and HCT116 cells in comparison to normal fibroblasts (Yamamoto et al., 2020a). We recently observed that high-malignancy variants of parental cancer cells have increased their methionine addiction, increased their extent of trimethylation of histone H3 lysine marks (Yamamoto et al., 2022), further emphasizing the association of their characteristics of cancer. Thus, there is strong association between methionine addiction, hypermethylation of histone H3 lysine marks, and malignancy. The apparent lack of global gene expression changes in the isogenic parent-and-revertant pairs suggest that rather than large scale ones, specific changes need to be investigated in the future, which may be critical for the malignancy to occur.

Methionine restriction alters the expression of certain genes. We and other investigators have previously reported that MR increased TNF-related apoptosis-induced ligand receptor-2 (TRAIL-R2) expression in cancer cells and enhanced the efficacy of TRAIL-R2 targeted therapy (Strekalova et al., 2015; Yamamoto et al., 2020b). However, the present study shows that there are few significant changes identified between parental cancer cells and methionine-independent revertants in global RNA expression, which is surprising given the large reduction of histone H3 lysine marks that are thought to be master regulators of gene expression. These results indicate that the loss of tumorigenicity in methionine-independent revertants may be because of translational and posttranslational controls. Further proteomic studies are needed to investigate translational and posttranslational controls in methionine-addicted cancer cells and their revertants.

In the present study, we did not detect a difference of the expression level of histone methyltransferases between methionine-addicted cancer cells and methionine-independent revertants by immunoblotting or RNA expression. In addition, the MAT2A protein level is not changed in the revertants. MAT2A is the major SAM synthase and is being targeted with candidate chemotherapy agents (Kalev et al., 2021). There are previous studies which report that high expression levels of histone lysine methyltransferases and MAT2A are markers of malignancy (Fei et al., 2015; Tu et al., 2018; Wang et al., 2019). However, our present study shows that methionine-independent revertants lose malignancy without significant changes in the expression levels of those enzymes. Methionine-pathway flux will be investigated by metabolomic studies in the future.

In addition, important is the previous finding by Breillout et al. that as cancer cells become more malignant as they become more methionine addicted (Breillout et al., 1990). Another important observation is from PET imaging of cancer in patients which consistently demonstrates that [^11^C] methionine gives a much stronger PET signal than [^18^F] deoxyglucose in head-to-head comparisons, demonstrating that cancers are methionine addicted in patients and that the Hoffman effect of methionine addiction is stronger than the Warburg-effect (Mitamura et al., 2018; Pirotte et al., 2004). Other molecules, such as various RNAs may also be overmethylated in cancer cells (Birnstiel et al., 1963; Tisdale, 1980). Further experiments are necessary to account for all the transferred methyl groups in methionine-addicted cancers and the differences in comparison of revertant with normal cells.

**Figure 4:**
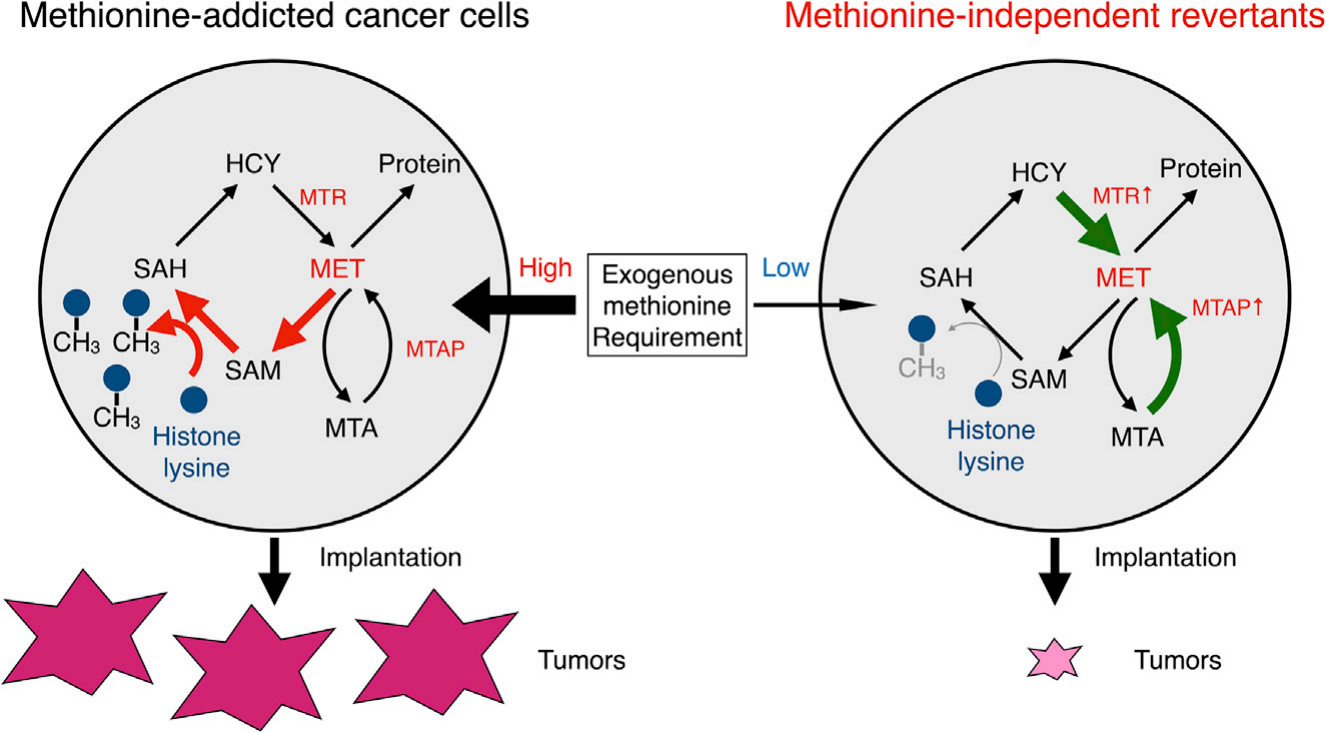
Properties of methionine-addicted cancer cells and methionine-independent revertants

The present results demonstrate that hypermethylation of histone H3 lysines is closely associated with methionine addiction and malignancy of cancer cells and possibly necessary for both (Figure 4). Methionine addiction is being widely recognized as critical for malignancy (Kanarek et al., 2020), as shown in the present study. Methionine addiction provides a potential universal target for methionine-restriction cancer therapy, such as with methioninase which depletes methionine in tumors rapidly and shown to be broadly effective against all cancer types tested, including patient-derived orthotopic-xenograft (PDOX) mouse models and in the clinic (Han et al., 2020; Kawaguchi et al., 2018a).

As mentioned above, we have previously published that under methionine restriction by methioninase, H3K4me3 and H3K9me3 methylation is strongly reduced in methionine-addicted HCT116 and H460 cancer cells, during which their growth is arrested and viability reduced (Yamamoto et al., 2020a). These results suggest that hypermethylation of these marks is necessary for the proliferation of the methionine-addicted cancer cells. These results are in contrast to normal fibroblasts which have reduced amounts of these marks, but they are stable under methionine restriction which still allows normal cells to grow. Raboni et al. have confirmed our observation showing methioninase treatment of the methionine-addicted cancer cell line caused a loss of the methylation of histone H3 lysine marks and arrested the growth of the cancer cells (Raboni et al., 2021).

Our model is that elevated methylation of histone marks such as H3K4me3 and H3K9me3 may be growth signals. It is very interesting that H3K4me3 becomes greatly elevated when T cells are activated to proliferate which greatly increases their methionine requirement, but is rapidly reduced under methionine restriction that arrests their growth (Bian et al., 2020; Roy et al., 2020; Sinclair et al., 2019), a marked similarity to methionine-addicted cancer cells, suggesting the possibility that these marks are growth signals. The multiple myeloma SET domain histone methyltransferase decreases H3K36me3 and suppresses growth of multiple myeloma cells (Martinez-Garcia et al., 2011). Future studies will further investigate the critical genomic changes associated with reversion of methionine-addicted cancer cells to methionine-independence and loss of malignancy and explore how such changes are related to loss of trimethylated histone H3 marks.

### Limitations of the study

The present study has shown the linkage between methionine addiction, histone H3 lysine hypermethylation, and malignancy. However, a causal effect between these phenomena has not yet been determined and will be the subject of further studies.

## STAR★Methods

### Key resources table

**Table:** 

REAGENT or RESOURCE	SOURCE	IDENTIFIER
**Antibodies**		

Anti-MAT2A	Novus Biologicals	Cat# NB110-94158SS; RRID:AB_1237163
Anti-AHCY	Proteintech	Cat# 10757-2-AP; RRID:AB_2289488
Anti-MTR	Proteintech	Cat# 25896-1-AP; RRID:AB_2880287
Anti-AMD1	Proteintech	Cat# 11052-1-AP; RRID:AB_2226430
Anti-MTAP	Abcam	Cat# ab126770; RRID:AB_11129069
Anti-H3K4me3	Cell Signaling Technology	Cat# 9751; RRID:AB_2616028
Anti-H3K9me3	Cell Signaling Technology	Cat# 13969; RRID:AB_2798355
Anti-H3K27me3	Cell Signaling Technology	Cat# 9733; RRID:AB_2616029
Anti-H3K36me3	Cell Signaling Technology	Cat# 4909; RRID:AB_1950412
Anti-H3K79me3	Cell Signaling Technology	Cat# 74073; RRID:AB_2799849
Anti-pan methyl lysine	Abcam	Cat# ab7315; RRID:AB_305840
Anti-total histone H3	Proteintech	Cat# 17168-1-AP; RRID:AB_2716755
Anti-SETDB1	Proteintech	Cat# 11231-1-AP; RRID:AB_2186069
Anti-SUV39h1	Proteintech	Cat# 10574-1-AP; RRID:AB_2196710
Anti-EHMT2	Proteintech	Cat# 66689-1-Ig; RRID:AB_2882043
Anti-GAPDH	Proteintech	Cat# 60004-1-Ig; RRID:AB_2107436
Anti-Ki-67	Proteintech	Cat# 27309-1-AP; RRID:AB_2756525
HRP-conjugated anti-mouse IgG	Proteintech	Cat# SA00001-1; RRID:AB_2722565
HRP-conjugated anti-rabbit IgG	Proteintech	Cat# SA00001-2; RRID:AB_2722564

**Chemicals, peptides, and recombinant proteins**		

L-homocysteine	Sigma-Aldrich	#69453
5′-Deoxy-5’-(methylthio)adenosine	Sigma-Aldrich	D5011
Fluoraldehyde o-Phthaldialdehyde Reagent Solution (OPA)	Thermo Fisher Scientific	#26025

**Critical commercial assays**		

EpiQuik Total Histone Extraction Kit	Epigentek	#OP-0006
Cell Counting Kit-8	Dojindo	CK04
Histofine SAB-PO® kit	Nichirei	03AM0770
Clarity Western ECL Substrate	Bio-Rad	#1705060

**Experimental models: Cell lines**		

HCT 116	ATCC	CCL-247
H460	ATCC	HTB-177
HCT 116-GFP	AntiCancer Inc.	N/A

**Experimental models: Organisms/strains**		

4-6 weeks old athymic *nu/nu* female mice	AntiCancer Inc.	N/A

**Software and algorithms**		

JMP PRO ver. 15.0.0	SAS	https://www.jmp.com/en_us/home.html
ImageJ ver. 1.53k	NIH	https://imagej.nih.gov/ij/index.html
Microsoft Excel for Mac 2019 ver. 16.58	Microsoft	https://www.office.com/
Photoshop Elements 2018 ver.16	Adobe	https://www.adobe.com/

### Resource availability

#### Lead contact

Further information and requests for resources and reagents should be directed to and will be fulfilled by the lead contact, Robert M. Hoffman (all@anticancer.com).

#### Materials availability

Recombinant methioninase is available upon request from the lead contact, Robert M. Hoffman (all@anticancer.com).

### Experimental model and subject details

#### Cell lines

The HCT 116 human colon cancer cell line and H460 human lung cancer cell line were used in the present study. HCT 116 cells were stably transduced to express green fluorescent protein (GFP) as previously described (Bouvet et al., 2006). HCT 116 and H460 cells were obtained from the American Type Culture Collection (Manassas, VA, USA) and were routinely checked for mycoplasma. Cells were maintained in DMEM supplemented with 10% fetal bovine serum (FBS) and 100 IU/ml penicillin/streptomycin.

#### Mouse studies

4-6 weeks old athymic *nu/nu* female mice (AntiCancer Inc, San Diego, CA, USA), were used in this study. All mice were kept in a barrier facility on a high efficacy particulate air (HEPA)-filtered rack under standard conditions of 12 h light/dark cycles. Animal studies were performed with an AntiCancer Institutional Animal Care and Use Committee (IACUC)-protocol specially approved for this study and in accordance with the principles and procedures outlined in the National Institutes of Health Guide for the Care and Use of Animals under Assurance Number A3873-1 (Yamamoto et al., 2020b).

### Method details

#### Recombinant methioninase production

Recombinant L-methionine α-deamino-γ-mercapto-methane lyase (rMETase) is a 172-kDa molecular homotetrameric PLP enzyme from *Pseudomonas putida* (Kudou et al., 2007). The rMETase gene was cloned in *E.coli*. rMETase-recombinant *E. coli* was fermented and rMETase was purified by a 50°C heat step, polyethylene glycol precipitation and DEAE Sepharose FF (Pharmacia, Uppsala, Sweden), column chromatography (Tan et al., 1997).

#### Selection and establishment of methionine-independent revertant cancer cells

The H460 and HCT 116 parental cell lines were cultured in normal methionine-containing medium with 1 U/mL of rMETase for more than one month as a first selection and surviving cells were isolated. These surviving cells were cultured in normal methionine-containing medium with 5 U/mL rMETase for more than 2 weeks as a second selection and surviving cells were isolated. The surviving cells isolated after the second selection were termed H460-R and HCT 116-R respectively (Figure 1A).

#### rMETase activity assay *in vitro*

Medium with 1 U/mL of rMETase was incubated. The fluoraldehyde *o*-phthaldialdehyde (OPA) reagent solution was added to the medium and derivatizes the amino acid in the medium. The OPA derivatized samples were loaded immediately on the HPLC column (Hitachi L-6200A Intelligent pump; Hitachi, Ltd., Tokyo, Japan) and methionine levels in the medium were measured, as an indicator of rMETase activity (Sun et al., 2005).

#### Efficacy of MR, effected by rMETase, on cell proliferation

Cells were cultured in 96-well plates (1 × 10^3^ cells/well) in normal DMEM overnight. The next day, the medium was changed to normal DMEM or DMEM with rMETase (1 U/mL). Cell proliferation was measured using the Cell Counting Kit-8 (Dojindo, Kumamoto, Japan) after 0, 24, 48, 72 and 96 hours of medium change.

#### Cell proliferation of methionine-independent revertatnts compared to parental methionine-addicted cancer cells in methionine-free medium containing homocysteine or methylthioadenosine (MTA)

Cells were cultured in 96-well plates (1 × 10^3^ cells/well) in normal DMEM overnight. The next day, the medium was changed to methionine-free medium containing 200 μM DL-homocysteine (Sigma) or 250 nM methylthioadenosine (MTA, Sigma) with 10% dialyzed fetal bovine serum. Cell proliferation was measured using the Cell Counting Kit-8 (Dojindo, Kumamoto, Japan) after 0, 1, 3, 5 and 7 days of medium change.

#### Immunoblotting

Extraction of histone and protein from cells and tumors and immunoblotting were performed as described (Yamamoto et al., 2020a, 2020b). Immunoreactive proteins were visualized with Clarity Western ECL Substrate (Bio-Rad Laboratories, Hercules, CA, USA). The UVP ChemStudio (Analytik Jena US LLC, Upland, CA, USA) was used to detect the signals.

#### Comparison of *in vivo* tumorgenicity of methionine-addicted parental cancer cells and methionine-independent revertants in a subcutaneous-tumor mouse model

Two different doses of HCT 116 and HCT 116-R cells (5 × 10^5^ or 1 × 10^6^ cells/100 μl PBS each) were injected subcutaneously into the flanks of nude mice. Each group comprised ten mice. The mice with HCT 116 or HCT 116-R tumors were sacrificed on day 28 and tumor weight was measured at termination (Yamamoto et al., 2021).

#### Comparison of ability of methionine-addicted parental cancer cells and methionine-independent revertants to form experimental liver-metastasis

Nude mice were anesthetized and HCT 116-GFP or HCT 116-R-GFP (1 × 10^6^ cells/100 μl PBS) were injected to the spleen of the mice during open laparotomy (Bouvet et al., 2006). Each group comprised three mice. The mice were sacrificed on day 42 and fluorescence intensity was measured at termination with the UVP ChemStudio (Analytik Jena US LLC) to detect the experimental metastasis.

#### H&E staining and immunohistochemistry

All tumors were resected and immediately fixed in 10% formalin. The resected tumors were embedded in paraffin. Tissue sections (4 μm) were made, and deparaffinized in xylene and rehydrated in an ethanol series. Hematoxylin and eosin (H&E) staining was performed according to standard protocols (Yamamoto et al., 2020b).

Immunohistochemistry was performed as follows: Tissue sections (4 μm) were heated in citrate-acid buffer (10 mmol/L citric acid, pH 6.0) with an autoclave. Then, endogenous peroxidase was blocked by 0.3% hydrogen peroxide in methanol. A primary anti-Ki-67 antibody (1:16,000, 27309-1-AP, Proteintech) was added to the tissue sections on slides and were incubated at 4°C overnight. The labeled antigens were stained with substrate-chromogen 3,3-diaminobenzidine (Dako, Glostrup, Denmark). Finally, these sections were counterstained in hematoxylin. The slides were observed with a model BH2 microscope (Olympus Corp., Tokyo, Japan).

For immunohistological evaluation, two investigators (K.H. and Y.A.) selected the five most abundant microscopic fields of each tissue and counted Ki-67-positive cells (magnification, 400×) in each of the five regions.

#### Next generation sequencing

Cells (1 × 10^6^) were pelleted and frozen at -80°C and provided to GENEWIZ (South Plainfield, NJ, USA) for RNA sequencing. Total RNA was extracted using a Qiagen RNeasy Plus Universal mini kit (Qiagen, Hilden, Germany). RNA sequencing libraries were prepared using the NEBNext Ultra II RNA Library Prep Kit for Illumina (NEB, Ipswich, MA, USA). The sequencing libraries were clustered on 2 flowcell lanes. After clustering, the flowcell was loaded on the Illumina HiSeq 4000 (Illumina, San Diego, CA, USA). The samples were sequenced using a 2x150bp Paired End (PE) configuration. One mismatch was allowed for index sequence identification.

### Quantification and statistical analysis

#### Statistics

All statistical analyses were performed with JMP PRO ver. 15.0.0 (SAS Institute, Cary, NC, USA). The Student *t*-test was used for comparison between groups. All data are expressed as mean ± SD or SEM of three independent experiments. A probability value of p < 0.05 is defined as statistically significant. ∗ p < 0.05, ∗∗ p < 0.01, ∗∗∗ p < 0.001.

## Data Availability

• All data produced in this study are included in the published article and its supplemental information, or are available from the lead contact upon request. • This paper does not report original code. • Any additional information required to reanalyze the data reported in this paper is available from the lead contact upon request.

## Supplemental information

Table S1. Genes with expression levels that are significantly different between methionine-addicted cancer cells and methionine-independent revertants in HCT 116, related to Table 1

Table S2. Genes with expression levels that are significantly different between methionine-addicted cancer cells and methionine-independent revertants in H460, related to Table 1

Table S3. Expression of genes related to protein methyltransferases in methionine-addicted cancer cells and their methionine-independent revertants, related to Table 1

Table S4. Expression of the genes for methyltransferases, other than protein methyltransferases, in methionine-addicted cancer cells and their methionine-independent revertants, related to Table 1
